# Patient Centricity in Patient Preference Studies: The Patient Perspective

**DOI:** 10.3389/fmed.2020.00093

**Published:** 2020-03-20

**Authors:** Eline van Overbeeke, Inès Vanbinst, Aura Cecilia Jimenez-Moreno, Isabelle Huys

**Affiliations:** ^1^Clinical Pharmacology and Pharmacotherapy, University of Leuven, Leuven, Belgium; ^2^Institute of Neuroscience, Newcastle University, Newcastle upon Tyne, United Kingdom

**Keywords:** patient involvement, patient participation, patient engagement, patient preferences, patient perspectives

## Abstract

**Objectives:** A factor contributing to the value of patient preference studies is patient centricity. This study aimed to explore how patients want to be involved in the design and conduct of patient preference studies. In addition, we investigated patients' expectations regarding the communication of study results back to patients.

**Methods:** Semi-structured interviews were conducted with patient representatives within three different disease areas: rheumatic diseases, cancer, and neuromuscular disorders. For each disease area, interviews were conducted with interviewees from Belgium, the Netherlands and the United Kingdom. Interviews followed a predefined interview guide covering topics relating to timing, level, and requirements for patient involvement in patient preference studies, as well as communication of results. Interviews were audio-recorded, transcribed and analyzed using framework analysis in NVivo 12.

**Results:** A total of 14 interviews were conducted. Some interviewees believed that patients should be involved in all steps of a patient preference study. Patient involvement seemed most valuable during the design phase to support defining research questions and instrument design. During analysis, patients can be involved for optimal interpretation of results. Most interviewees mentioned that patient involvement should be on the level of advice or collaboration, not control. Interviewees expressed requirements for patient involvement relating to the knowledge of the involved patient, time investment, compensation and other incentives. Regarding communication of results, most interviewees wished to receive a brief and lay summary of the results, followed by a detailed explanation of both individual and average results accompanied by visuals.

**Conclusions:** Patient involvement in patient preference studies could increase question comprehension by study participants and ensure correct interpretation of results by researchers. Patients want to be involved as advisors or collaborators, and considering their personal situation as well as establishing agreements on roles, time involvement and compensation early on will result in a most optimal partnership.

## Highlights

- Patients can be involved in defining research questions, as well as questions, attributes and levels for patient preference studies
- Patient involvement could lead to more optimal interpretation of patient preference study results
- Patients appreciate to be involved as advisors or collaborators
- Enough educational and compensational support should be provided to allow a fair involvement
- Patients wish to be involved through multiple short sessions

## Introduction

The health care system is undergoing a paradigmatic shift and the role of patients is evolving from being passive recipients to becoming autonomous and actively involved participants (1). Patients are increasingly involved in healthcare decision-making at the micro level, also referred to as individual patient involvement or shared-decision making (2, 3). Experiential knowledge, the kind of expertise that is gained by patients through experiencing illnesses and therapies, is increasingly considered complementary to health care professionals' expertise (4, 5). Beyond decisions related to their personal healthcare, patients are also increasingly requested to take on active roles in drug development, e.g., provide input in clinical trial design, scientific advice procedures, discussions with regulators and health care policy decision-making at the meso and macro level; also referred to as collective patient involvement (3–7). Abma and Broerse (8) defined different levels of patient involvement from consulting as study subject to obtaining control as client.

While collective patient involvement increasingly occurs, this direct form of patient involvement is criticized by some as doubt exists on whether one patient or a small number of patients can represent the views of the majority (9, 10). Patient preference studies explore, measure and assess treatment preferences of patients and are a tool to incorporate the voice of a larger group of patients in the medical product life cycle (e.g., in value assessments like benefit-risk assessment and health technology assessment). Use of patient preferences in itself can be seen as a form of indirect patient involvement (10). The Patient Preferences in Benefit-Risk Assessments during the Drug Life Cycle (PREFER) project aims “*to strengthen patient-centric decision-making throughout the life cycle of medicinal treatments by developing expert and evidence-based recommendations on how patient preferences should be assessed and inform decision-making”* (11). A factor contributing to the value of patient preference studies is patient centricity, referring to the extent to which patients are involved in the design and conduct of these studies (12–14). The Food and Drug Administration (FDA) also recognized this and stated in their guidance on patient preference information (15) that the patient should be “*the central focus of the study.”*

The European Patients' Academy (EUPATI) created, together with patients, guidance on patient involvement in development, ethical review of clinical trials, regulatory evaluation and health technology assessment of drugs (6, 7, 16–18). While guidance exists on patient involvement in these processes, it remains unknown how to involve patients in patient preference studies. The aim of this study was to explore how and when patients want to be involved along the design and conduct of patient preference studies, as well as to understand the expectations of patients regarding communication of study results back to participants.

## Methods

### Population

Semi-structured interviews were conducted with patient representatives within three different disease areas: rheumatic diseases (RD), cancer (CA), and neuromuscular disorders (NMD). Patient representatives had been involved in activities of patient organizations or patient preference research, and were either patients themselves or caregivers to a patient. Caregivers were defined as parent(s), legal guardian, or other adult family member living in the same house or in contact with the patient in a caregiver relationship at least 4 times/weeks for at least 1 h or more, and were only included in the sample if the patient could not participate in the interview due to age (pediatric patients) or cognitive impairments. The three disease areas selected (i.e., RD, CA, and NMD) represent diseases that vary not only by disease phenotype but also in prevalence, chronicity, and medical unmet needs. For each disease area, interviews were conducted with interviewees from Belgium, the Netherlands, and the United Kingdom (UK). These three countries were selected as they cover different patient involvement cultures. While the Netherlands has a highly developed patient involvement model (19), the UK has less formalized processes but is still reaching high patient involvement (20), and Belgium has low and scattered patient involvement (10).

### Interview Guide

An interview guide was established (Supplemental Material I) based on the findings of previous PREFER studies that highlighted the need for patient involvement in the different phases of patient preference studies (12, 13), and on levels of patient involvement as discussed by Abma and Broerse (8). The interview guide consisted of parts relating to (1) interviewees' experience and knowledge on patient involvement and patient preference studies, (2) patient involvement in design and conduct of patient preference studies, and (3) communication of results to patients. The guide was written in lay language and was piloted in an interview with a representative of EUPATI Belgium. To prevent the interview guide from becoming disease-specific, no patients from the target populations were involved in the design. The interview guide was subsequently translated from English to Dutch for interviews in Belgium and the Netherlands, allowing interviewees to express themselves in their native language.

### Participant Recruitment

Interviewees were recruited via mail through purposive sampling and snowballing using the network of EUPATI Belgium and PREFER. In purposive sampling, interviewees are chosen based on their ability to provide insights regarding the study purpose (21). In this study, patient representatives were chosen as subjects because of their expertise in their disease and patient involvement or patient preferences. The goal was to interview at least one, preferably two, patient representatives per disease category per country.

### Conduct

Semi-structured interviews were executed in person or through teleconference. During the interviews, the interview guide was used to present an example of a patient preference study specific to the disease area of the patient representative, and to ask predetermined questions. However, open discussion was also encouraged to explore opinions in-depth. After informed consent was given, a short demographics and health literacy (22) questionnaire was completed. Interviews were audio-recorded and then transcribed verbatim. All the transcripts of the interviews were produced in the original language and non-English quotes were only translated to English upon inclusion in the paper.

### Analysis

The transcripts were analyzed using *NVivo 12* following framework analysis, a type of thematic analysis (23) (Supplemental Material II). The analysis started with a familiarization process, including conducting, transcribing and reading interviews, to ensure that researchers obtained an overview of the dataset. Themes of the interview guide informed the creation of deductive codes. The first 4 transcripts were independently coded by two researchers (IV and EvO) and then compared. Based on observed patterns and critical observations inductive codes were created. The inductive and deductive codes together formed a “coding tree.” The coding tree was uploaded in NVivo and applied to all transcripts, where sections of transcripts relating to a particular theme were classified under the respective code. All data were summarized into a framework matrix. The data of the interviews were interpreted, summarized per code, and some quotes of individual interviewees were added for clarification.

## Results

In total 14 interviews were conducted (Table 1). More female than male patient representatives and no adolescents were recruited. Almost all interviewees were patients themselves; only one NMD caregiver was recruited in the Netherlands. The sample included one or more patient representatives per disease area per country and most interviewees had adequate health literacy (i.e., a health literacy score of <2). Results of the interviews are described below per theme. Codes following quotations refer to interviewees' characteristics: BE, Belgium; CA, cancer; NL, the Netherlands; NMD, neuromuscular disorders; RD, rheumatic diseases; UK, United Kingdom.

**Table 1:** Demographics.

**Characteristics**	**Interviewees (*****n*** **=** **14)**	
	***n***	**%**
**Sex**		
Females	9	64,29
Males	5	35,71
**Age, Years**		
18–24	0	0
25–39	3	21,43
40–60	9	64,29
>60	2	14,29
**Country**		
United Kingdom	4	28,57
Netherlands	3	21,43
Belgium	7	50
**Disease Area**		
Neuromuscular disorders	5	35,71
Rheumatic diseases	5	35,71
Cancer	4	28,57
**Stakeholder Group**		
Patient	13	92,86
Caregiver	1	7,14
**Years Since Diagnosis**		
<1	0	0
1–3	1	7,14
4–10	6	42,86
>10	6	42,86
**Health Literacy**		
Adequate	11	78,57
Inadequate	3	21,43

### The Meaning of the Term “Patient Preferences” to Patients

Patients were asked to explain their understanding of the term “patient preferences.” Interviewees described patient preferences as what patients find important in their experience of the illness, how they experience their quality of life and what is needed to improve that quality. They expressed that patient preferences are very individual. In addition, they stated that patient preferences represent the right to have the choice to accept or refuse treatments. “*Preferences should be taken into account during all parts of the journey: from diagnosis right through the stages of treatment and side effects”* (CA_UK_13). It was stated that patient preferences are also about developing drugs in a way that patients want them to be developed. A UK cancer interviewee emphasized that not only patients' preferences, but also the partner and family's preferences should be considered.

When the definition of patient preferences and the example of a patient preference study were presented to the interviewees, all 14 interviewees understood that patient preferences cover health treatment choices, and that a comparison and preference is made based on received information covering the different options. One suggested the term “*treatment preferences”* as a possible synonym for patient preferences (CA_NL_14).

### Patient Involvement Along the Phases of Patient Preference Studies

**Figure 1:**
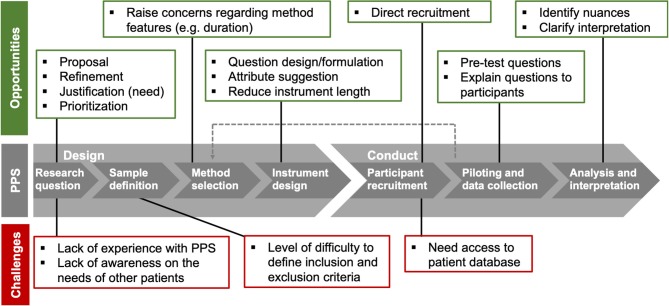
Overview of opportunities and challenges for patient involvement in patient preference studies (PPS). Opportunities (green) and challenges (red) were identified in interviews with patient representatives.

Interviewees were asked in which steps of a patient preference study they thought their input as a patient could be of most value and why. Opportunities and challenges for patient involvement along the different phases of patient preference studies were identified (Figure 1). According to five interviewees, patients should be involved in all steps of a patient preference study. Involvement of patients was especially found valuable in the set-up of the study. Involving patients early-on would also allow patients to be aware of every aspect and decision made in the study.

#### Defining Research Questions

Having patients involved in defining research questions was found important by five interviewees. Involving people from the start, gives patients and researchers the opportunity to discuss the need and initial considerations for the study. Patients can be involved in defining research questions by either proposing them, or by providing suggestions to refine those proposed by researchers. Three interviewees from Belgium thought that it would be difficult for patients to define research questions for patient preference studies, since (1) experience with this kind of studies is required and a lot of patients are not aware of the existence of patient preference studies, and (2) patients should be aware of needs of the full patient population to propose relevant research questions. Interviewees agreed that there is value in involving patients in the prioritization and refinement of research questions based on their unmet needs; “*Having life experience always helps to filter out ideas”* (NMD_UK_6). A benefit of involving patients in defining research questions, as suggested by interviewees, was that it could prevent misunderstandings in further phases of the study.

#### Sample Definition

While half of the interviewees mentioned that patients could be involved in defining the patient sample, they considered professionals to be better placed than patients to make these decisions. Two Belgian interviewees explicitly stated that the patient sample should not be defined by patients. One interviewee explained that it is difficult for patients to define inclusion and exclusion criteria. On the other hand, another interviewee felt that defining the target patient population should be “*quite clear*” (RD_BE_1).

#### Preference Method Selection

Two interviewees mentioned that patients could be involved in the selection of preference exploration or elicitation methods. They believed it was valuable to discuss with patients how preferences should be measured to address concerns regarding features of these methods, like the duration of a survey.

#### Instrument Design

Ten interviewees elaborated further on involving patients in the design of questions, attributes, and levels. They believed patients could help design or formulate the questions. Interviewees recognized that formulation is important and has to be checked with patients as small nuances could result in study participants having “*different degrees of how they understand things and interpret things”* (NMD_UK_2). A cancer patient from the Netherlands stated that patients are increasingly being involved in the design of questions for interviews and surveys. Interviewees also mentioned that based on the patients' experience with treatments that they can suggest inclusion of attributes relating to treatment (side) effects that professionals may have not considered important. Involving patients in the design of instruments could, according to interviewees, lead to the creation of surveys of reasonable length; to prevent researchers from asking too much at once, e.g., “*we will measure this too, and this seems also interesting”* (RD_BE_3), and to prevent patients from getting tired, losing their concentration, and providing inaccurate answers.

#### Participant Recruitment

Seven interviewees felt that patient organizations, not individual patients, could play an essential role in recruiting participants for patient preference studies, as they often have direct access to patient databases. Patient organizations could provide “*access and information”* (CA_NL_14) to the study on their website or via brochures. Furthermore, patients could also try to directly convince and “*motivate”* (RD_NL_8) others in their environment to participate in patient preference studies. Interviewees expressed that patients are more likely to be convinced to participate in studies “*by fellow patients”* (RD_BE_3) or “*experience experts”* (RD_BE_5) than “*by a researcher who sends yet another email or letter”* (RD_BE_3).

#### Data Collection

Seven interviewees shared the opinion that collecting data is not particularly a step where patients could be involved. Collecting preferences was seen as a task for researchers as it requires an “*analytical mindset”* (RD_BE_5). Nevertheless, five interviewees indicated that there was a role for patients during data collection. Two interviewees stated that participants in a patient preference study might not understand the questions asked to them and that a patient could accompany the researcher to comfort participants and explain the questions; “*patients might understand better if it's explained to them by somebody who knows what it's like to live with that disease”* (RD_UK_12). This was found particularly useful for specific populations like “*elderly”* or “*people with lower health literacy”* (CA_NL_14). It was suggested by two interviewees that patients could also be involved through pilot interviews to test questions.

#### Analysis and Interpretation of Results

Interviewees believed that the analysis of patient preference studies should be done by trained professionals. Nine interviewees shared the opinion that it would be valuable to involve patients in interpretation of results, to identify nuances. An NMD interviewee from Belgium stated that it could be difficult for researchers to interpret an answer without having experience with the disease context. A Belgian RD interviewee commented that interpretation can take place on multiple levels; ranging from looking at “*raw data”* trying to gain insights, to looking at processed results in order to clarify the interpretation. Two interviewees from Belgium did not find it necessary to involve patients in analysis and interpretation.

### Levels of Patient Involvement in Patient Preference Studies

**Figure 2:**
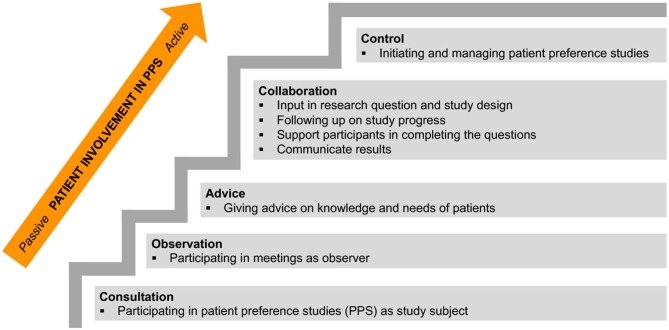
Staircase of patient involvement levels in patient preference studies (PPS). Roles of patients and patient involvement levels were identified through interviews and the patient participation model of Abma and Broerse (8), and are displayed from passive to active involvement.

The interviewees were asked what roles patients want to have in patient preference studies along different involvement levels (Figure 2). The four levels that were presented to interviewees were consultation, advice, collaboration, and control. An interviewee from the Netherlands mentioned a fifth level of patient involvement, based on own experience, where patients can act as observers in meetings to ensure voices are well-balanced. Six interviewees stated that patients can be involved in all levels. The level of involvement that a patient can take on seemed to depend on patient-related factors like “*stage of disease*” (RD_BE_3), but also study-related factors as described below.

#### Consultation

Half of the interviewees mentioned that a lot of patients would want to participate in patient preference studies as subjects. Interviewees argued that some patients might think they do not have enough knowledge and would be scared to take on a more involved role. A Dutch interviewee strongly felt that consultation as study subjects cannot be considered to be patient involvement and stated it is “*a necessity and not part of including patient's perspectives*” (NMD_NL_10).

#### Advice

Most of the interviewees felt that giving advice on the design and conduct of patient preference studies, was an adequate level of patient involvement. Interviewees mentioned that advising roles require “*a little preparation”* and that patients taking up these roles should be “*people who have more than just basic knowledge*” (RD_BE_1). Patients might advise researchers on “*what patients know*” (NMD_UK_2) and what their needs are. However, one interviewee argued that it is very difficult to find people capable of giving “*good advice”* (NMD_BE_9) as patients may have limited medical knowledge or may be desperate for treatments.

#### Collaboration

Almost every interviewee argued that patient involvement on a collaborative level, as research partners, would be very valuable. Working with researchers in a collaborative approach might involve patients actively “*brainstorm(ing) about the design and what should be investigated”* (RD_BE_1), giving input in decisions that need to be made to obtain “*relevant research”* (NMD_NL_10), having the opportunity “*to really follow things up”* in order to “*share it afterwards”* (RD_BE_5), and “*work(ing) with the participants”* (RD_UK_12) by helping other patients to complete a survey or interview. It was suggested to find people suited for this role via patient organizations. Two interviewees stressed that patients need to have the time and energy to collaborate; “*patients are volunteers and some patients are not well enough to commit fulltime”* (CA_UK_13). A Belgian interviewee emphasized the importance of giving collaborating patients “*sufficient support”* (RD_BE_5).

#### Control

It was advised by the majority of interviewees not to put patients in control of patient preference studies, but to leave this to trained researchers. They felt that it cannot be expected from patients to be aware of all ongoing scientific research and that reversing the roles would be less effective. Three interviewees mentioned that patient organizations possess the know-how and could potentially be in control of patient preference studies.

### Requirements for Patient Involvement in Patient Preference Studies

Interviewees were asked about requirements for patient involvement relating to skills, time investment, incentives and barriers. Open communication and pre-defined arrangements regarding expectations, commitment, compensation and personal situation will, according to the interviewees, result in comfortable, and pleasant collaboration.

#### Skills

Interviewees mentioned skills that are required of patients involved as advisors or collaborators. These requirements included (1) “*sufficient mental capacities”* (NMD_BE_11), (2) disease knowledge, (3) experience with research, (4) familiarity with “*the jargon”* (CA_NL_14), (5) drive and motivation, (6) skills in the “*political, strategic game”* (NMD_NL_10), and (7) capable of “*transcending their own disease situation”* (RD_BE_3) to be aware of the perspective of the whole patient population. A UK interviewee felt that patients who are part of patient organizations, are automatically a “*certain type of persons”* (RD_UK_12) and can easily be involved in design or conduct of patient preference studies. Three interviewees shared the opinion that “*a variety would be good”* (NMD_UK_2); having a combination of patients with and without experience.

#### Time Investment

In general, interviewees stated that there are definitely patients willing to invest time in patient preference studies. The time and energy that patients would be willing to invest depends, according to the interviewees, on different factors. Patient-related factors seemed to include (1) work situation, (2) family circumstances, (3) treatment plan, and (4) personal interests and expectations. Study-related factors included (1) provision of financial compensation, (2) flexibility, and (3) the desired level of patient involvement. Interviewees estimated a reasonable time investment for patients to be “*one to 2 h a week”* (RD_BE_1) to half a day per week, and stated that the number of consecutive hours that patients are requested to be involved should be limited to 3 h. In general, interviewees preferred multiple short durations of involvement over long consecutive periods. Two interviewees were willing to “*give as much time as needed to this cause”* (NMD_UK_6). All interviewees expressed the importance of having expectations agreed upon beforehand and that time should be used efficiently.

#### Incentives and Barriers

Almost every interviewee showed willingness to be involved in design and conduct of patient preference studies. Four interviewees did not need any incentives, and argued that the value of patient preference studies for future drug development would be sufficient. Barriers that could impede patient involvement were: (1) too much time and energy asked from patients, (2) difficult accessibility of research meetings, (3) the use of technical medical terms, and (4) patients' physical condition and treatment plan. Incentives of interest to patients included: (1) patient-relevant study objectives, (2) good collaboration between all stakeholders, (3) extensive briefing on the purpose of the study, and expectations and timing of the involvement, and (4) providing the possibility to complete tasks from their own homes “*in the time that suits them”* (RD_UK_12) and attend meetings online, (5) financial compensation, and (6) sufficient support from the research team. Regarding financial compensation, seven interviewees stated that the expenses (e.g., travel expenses) resulting from their involvement should be covered. Financial incentives that go beyond reimbursement of travel expenses, would be “*a definite enticement”* (RD_UK_12) and were mentioned as a requirement by three interviewees. Two interviewees argued that patients should be financially compensated in proportion to the time and energy invested. A Dutch cancer interviewee insisted that compensating the time invested by patients shows that their involvement is taken seriously by the research team. Other incentives like vouchers, provision of alimentation, co-authorship on a publication, and arranged transport were suggested as alternatives for financial compensation.

### Communication of Patient Preference Study Results to Patients

The final theme discussed with all interviewees was on how to communicate patient preference study results to patients. First, the value of communicating study results to patients was discussed, followed by how results should be presented to patients. Patients wanted to receive results for their own education and interest. The majority of interviewees would want to receive a brief lay summary of the results, followed by a detailed explanation to have the opportunity to explore the results in depth. Interviewees stated that results should be “*reduced to the essence”* (NMD_BE_9), without leaving out important results. Eleven interviewees preferred text to be accompanied by graphs or other visuals. In addition, almost every interviewee wanted to receive both individual and average results, in a way that they would be able to compare their individual results with the group average. One interviewee questioned “*whether it would help”* (NMD_UK_2) to receive information on other patients' preferences, given that in some disease areas severity and symptoms of disease can vary largely between patients. A Belgian RD interviewee felt that it could be dangerous to compare individual results to the average, as it can be perceived as if participant's preferences are judged.

## Discussion

This qualitative study builds on results from previous research describing the value of patient centricity in patient preference studies and need for more research on how patients can be involved (12, 13). Therefore, the purpose of this study was to investigate how and when patients want to be involved in the design and conduct of patient preference studies. Roles, levels and requirements for patient involvement, as well as communication of results to patients, were discussed with patient representatives.

### Roles and Levels of Patient Involvement in Patient Preference Studies

According to interviewees, patient involvement in patient preference studies is most valuable when defining research questions, defining instruments (questions, attributes, levels) and interpreting results. Emphasis was placed on involving patients from the beginning to discuss unmet needs and refine research questions. A similar mindset can be observed in drug development and clinical trial design. Geissler et al. (24) discuss the vital role of patients at early stages of drug development, where priorities can be aligned with patients' unmet needs. Similarly, Crocker et al. (25) showed how early patient involvement in clinical trial design can prevent researchers from conducting trials that are not of value to patients. Involving patients in formulating questions, attributes and levels, for patient preference studies can ensure understanding by study participants. The FDA guidance on PPI explains that comprehension by study participants is important and a full understanding of harms, risks, benefits, and other communicated medical information is vital to obtain valid patient preference study results (15). Differently than what has been described about patient involvement in clinical trial design, interviewees pointed out the importance of including patients during the interpretation of patient preference study results (6, 24). This difference can be explained by the nature of data resulting from clinical trials and patient preference study. While results of clinical trials are clinical data and not much open for interpretation, results from patient preference studies are mostly based on subjective perceptions of treatment features. Involving patients when interpreting patient preference study results can provide insights into the reasons why patients answer in a certain way or give importance to certain features over others.

The majority of interviewees believed that patients should be involved in patient preference studies as advisors or collaborators, but not as the ones being in control. This result confirms the statement of Abma and Broerse that partnership between involved patients and researchers has been preferred over delegated power and citizen control (26). Moreover, our results show the importance of early agreement on the expected level of involvement and its implications. The EUPATI guidance document for R&D also states that the level of input should be discussed and agreed at start (6). In addition, our results show that not all patients can be involved at all levels and that agreements should reflect patients' personal requirements.

While interviewees in general seemed to agree on the roles and levels of patient involvement, some differences in attitudes toward patient involvement were identified. Some of these differences seemed to be associated with the interviewee's county of origin. Interviewees from the Netherlands believed in active patient involvement and had a clear view on what they perceived to be patient involvement (e.g., one patient representative proposed a fifth level of patient involvement, and another did not consider “consultation” to be patient involvement), while interviewees from Belgium were less confident. Belgian interviewees perceived barriers like limited knowledge and capacities of patients, and some did not think patients were able to define research questions and patient samples or provide valuable input in interpretation of results. These results show that patient involvement in patient preference studies can also be hampered by cultural beliefs.

### Requirements for Patient Involvement in Patient Preference Studies

Most of the interviewees believed that patients should be aware of the perspectives of a whole patient population, transcending their own disease, in order to take on highly involved roles in the design and conduct of patient preference studies. The interviewees tended to prefer patient advocates and patient experts of patient organizations over “lay patients” as candidates for involvement in patient preference studies. These results are not in line with the EUPATI guidance for patient involvement in industry led drug R&D that states that all types of patients should be involved (6). This difference may originate from a lack of confidence in “lay” patients regarding their knowledge on patient preference studies that could be improved with appropriate training and support from researchers. Harrison et al. (27) indicate that giving training to patients and researchers on the content of the study and on patient-researcher collaboration is an important “best practice activity”. From the heterogeneity of responses gathered in this study on roles and levels of patient involvement, it can also be concluded that it would be preferable to involve multiple patients (more than one individual) in patient preference studies as opinions on study design and conduct may also differ among patients.

Regarding the time that patients are willing to invest to be involved in patient preference studies, our results showed that this time differs between individuals and depends on various personal and study-related factors but that full-time or long consecutive hours would not be feasible. Interviewees with RD were most concerned about time constraints when combining work schedule and personal life. Cancer and NMD interviewees highlighted the impact of their treatments on physical well-being and the amount of energy left to dedicate to a study. If patients are not willing or able to be involved throughout the full process, the researchers propose to allow for flexible involvement whereby different patients individually take on different tasks.

On the topic of incentives, more than half of our interviewees wanted travel expenses to be covered. This confirms the statement of Harrison et al. (27) that reimbursement of travel expenses is an absolute minimum requirement for patient involvement in research. Only a few interviewees expressed the need for additional financial compensation depending on the time and energy invested by a patient. The EUPATI guidance for patient involvement in industry led drug R&D (6) also recommends to provide compensation for patients' total time investment in addition to expenses. Harrison et al. (27) agrees that compensating patients for their expertise and time besides covering travel expenses is good practice. Unfortunately, there is not always a budget for patient involvement, making financial compensation a remaining obstacle of patient involvement in research in general. Our results also confirmed patients' willingness to receive non-financial compensation as proposed in EUPATI's guidance (6).

### Communication of Patient Preference Study Results

In the current study interviewees were asked how results of patient preference studies should be communicated to patients. The interviewees placed emphasis on the use of lay language and visuals. Most interviewees wished to receive a brief and lay summary of the results, followed by a detailed explanation of both individual and average results accompanied by visuals. Wolka et al. (28) went beyond these general requirements and stated that the communication of these results should be tailored to the targeted patient population. While efforts should be made to communicate results of preference studies back to study participants, there may be barriers preventing this. These barriers may include (1) anonymous participation of patients in these studies, (2) the time that it takes to prepare results for dissemination, which may exceed study funds, investigators' availability or participants' expectations, and (3) legal barriers preventing industry sponsors from having direct contact with patients (10). In this case, we propose to provide a summary of sample-level results to recruiting parties (e.g., patient organizations and physicians) for them to share with their patients and support dissemination. Still, better strategies on how and when to communicate results back to study participants and other patients are needed.

### Strengths and Limitations

Although interviews by nature provide subjective evidence that may not be generalizable to other populations, our study design safeguarded the inclusion of diverse types of patients from three different disease areas across three countries, reducing population specificity.

A limitation of this study is the small patient sample included (i.e., 14 interviewees). Therefore, we should be cautious when extrapolating conclusions. During recruitment, we experienced low response rates that seemed to be related to the exhaustion of patient populations. Our overall pool of candidates was already limited to start with as we focused on experienced patient representatives. Active and more experienced patient representatives working for patient organizations are increasingly being contacted by researchers, resulting in difficulties to commit to new requests. In our study, out of the three disease areas, cancer patient representatives were the hardest to recruit. Another limitation is the lack of collaborative involvement of patients throughout design and conduct of the current study, caused by the small pool of candidates and lack of non-disease specific patient representatives. The researchers felt that involving patients from one or two of the investigated disease areas could have led to a disease-specific design, and would also have led to an even smaller sample of candidate participants. Therefore, the design was only discussed with a patient representative involved in EUPATI Belgium; a non-disease specific initiative promoting patient involvement through patient education. Due to high demand, the EUPATI BE patient representative was not available for further involvement in conduct and analysis stages of the study.

Analysis of health literacy questions showed that three interviewees had inadequate health literacy. However, the interviewers did not experience any difficulties when discussing patient preference studies with these interviewees. Therefore, the researchers believe that these health literacy scores did not affect the results of the interviews. Moreover, these results show that patients with inadequate health literacy are able to understand patient preference studies and therefore could potentially be involved in the design and conduct of these studies.

All interviews were conducted by the same interviewer (IV), reducing the variability between interviews. The interviewer was a junior researcher, but was supervised and trained by a researcher (EvO) with experience in conducting qualitative research, including the conduct of interviews and focus groups with patients. The analysis of the data was also done by these two researchers as they were most familiar with the dataset.

### Future Perspectives

This qualitative study provides insights on how to, according to patients, involve patients in the design and conduct of patient preference studies. Further qualitative studies could be set up to explore perspectives of other stakeholders, such as specialized health care professionals and researchers conducting patient preference studies, to understand how they want to involve patients and to provide additional insights on topics for which heterogeneity in responses was observed among patients (e.g., the involvement of patients in sample definition and data collection). Furthermore, quantification of our results in a larger patient sample could improve the generalizability of these results to a wider population and investigate heterogeneity in responses. Moreover, opinions from various stakeholders could be combined in recommendations on patient involvement in patient preference studies. As mentioned above, also strategies on how and when to communicate results back to study participants and other patients should be further explored.

## Conclusions

Patient involvement in patient preference studies is, according to patient representatives, most valuable in early and late stages of these studies; more specifically in defining research questions, formulating questions, attributes and levels, and during interpretation of results. Regarding communication of patient preference study results to patients, patients prefer the combination of a lay summary and a detailed report with visuals. Patients believe the best way to involve them in the design and conduct of patient preference studies would be as advisors or collaborators. Open communication and pre-defined arrangements regarding expectations, commitment, compensation, and personal situation are crucial. Considering these requirements in patient-researcher collaborations during future patient preference studies could ensure an optimal experience for patients.

## Data Availability Statement

The datasets generated for this study will not be made publicly available. Participants did not provide consent for the sharing of interview transcripts with parties other than the researchers. Requests to access these datasets should be directed to Eline van Overbeeke, eline.vanoverbeeke@kuleuven.be.

## Ethics Statement

All interviewees provided written informed consent prior to starting the interview. Ethical approval was obtained from all relevant ethics committees, including the Medical Ethics Committee of UZ KU Leuven/Research in Belgium (MP007215) and the Ethics Committee of the University of Newcastle (BH162126). The Central Committee on Research Involving Human Subjects (CCMO) of the Netherlands exempted the study from ethical review in the Netherlands.

## Author Contributions

EO and IV designed the interview guides. The interview guides were reviewed and approved by AJ-M and IH. Recruitment and planning of interviews were organized by EO, IV, and AJ-M. Interviews were conducted by IV and supervised by EO. The analysis was conducted by EO and IV. EO produced the first draft of the manuscript, which was subsequently revised and finalized with all authors. All authors approved the final manuscript.

### Conflict of Interest

The authors declare that the research was conducted in the absence of any commercial or financial relationships that could be construed as a potential conflict of interest.
