# Supplementary material for: Patient Centricity in Patient Preference Studies: The Patient Perspective
Source: Front Med (Lausanne). 2020 Mar 20;7:93. doi: 10.3389/fmed.2020.00093 (PMC7100374; doi:10.3389/fmed.2020.00093)
Supplement: Supplementary file 1 [file Data_Sheet_1.PDF]

Patient centricity in patient preference studies: the patient perspective
Supplemental material

Supplemental material I: Interview Guide

❖ Introduction

- *Present **yourself** (name, master thesis student)*
- ***Thank patient** in advance*
- *Check if **informed consent** form is signed*
- *Short explanation:*
    - *Interview will be **recorded in a few minutes***
    - *Interview will approximately take **30 min to 1 hour***
    - *Data will be **pseudonymized**; a code will be given to your information so that you won't be identifiable*
    - *There are **no wrong answers,** we want your open ideas/opinion*
    - *Participation is **voluntary**, you can withdraw at any point*
    - *You are **not obliged to answer** questions you don't feel comfortable about*

- *Background:*
    - ***Patient preference studies** are becoming increasingly important. In those studies, preferences are measured among different, alternative health interventions in a population sample of patients. Outcomes of these studies can be integrated in decision-making in the lifecycle of a medicinal product or device.*
    - *In **previous interviews** concerning this topic, it became clear that it would be **useful to involve patients** in the design and conduct of these preference studies, to ensure that participants fully understand harms, risks, benefits and other medical information. This interview tries to find answers on the question 'how' to involve patients.*

- *The goal of this interview (3 parts):*
    - *Some introductory questions*
    - *We want to know how patients can/want to be **involved in the design and conduct** of those patient preference studies*
    - *What are expectations regarding **communicating study results**.*

→ *is everything clear?*
→ *I am going to start the recording*

❖ Introductory questions

1. Can you tell me something about your **experiences in research** as a patient?

2. What is your **role** as a patient expert/advocate?

→ *we are going to discuss an important term for this interview*

3. **What do you think about** when you hear the term **‘patient preferences’**?

   (What does **‘patient preferences’ mean** to you?)

   "*Patient preferences reflect why patients choose a particular health treatment over other available options. This health treatment can be a drug or a medical device. To make a choice or state a preference, patients need to weigh up the advantages and disadvantages and compare them to those of other health treatments.*"

→ *Is this definition clear to you? Do you want to add something?*
→ *Before we start with the specific questions on patient preference studies (PPS) for this interview, I will explain a short example of a PPS just for your information, so it will be easier to answer the questions.*

❖ Case/Example of PPS

	**Rheumatic Disorders (RD)**	**Neuromuscular disorders (NMD)**	**Cancer (CA)**
**Reason for this PPS**	Early diagnosis is associated with a better outcome for the Rheumatic Arthritis (RA) patient.	For NMD, a group of rare diseases, not many has been accomplished yet to characterize what patients find important, what the most important attributes are for medical interventions.	A new combination therapy (immuno- and chemotherapy) for lung cancer patients, with better chances of survival, but also various side effects.
**Goal**	What are the preferences of ‘at risk’ individuals about preventive treatments for RA? (not yet diagnosed)	To identify and quantify the ‘unmet health priorities’ and preferences of NMD patients for different potential treatments (incl. benefit-risk trade-offs).	Discover what patient preferences are about the pros and cons of different treatment options in relation to the chances of survival of each treatment for lung cancer.
**Sample**	- First degree relatives of RA patients (4 times higher chance then general population)<br>- The general population (1/100)	- DM1 (myotonic dystrophy type 1) and MD (mitochondrial disorders) early onset diagnosed (before 20 years)<br>- DM1 and MD late onset diagnosed (after 20 years)<br>- Caregivers of a DM1 or MD patient	Stage IV lung cancer patients, who have experience with immunotherapy.
**Method**	- Focus groups (family members of RA patients and general population)<br>- Online surveys of 40 min (DCE or PTT) where they need to choose between different options	- Interviews and focus groups<br>- Online surveys (3 methods: DCE, BSW2, Q-methodology) where patients have to choose between different options	Interviews and focus groups (= discussions in small groups)

**Value of results**	- Results from the patient preference study will be valuable to inform development and regulation of efficient preventive strategies for RA.<br>- Interesting to compare preferences between countries, or between general population and family members of RA patient	- Results of the PPS can inform decision-making (e.g. Industry, regulators) about the 'unmet needs' of the NMD patient population, for the development of new treatments for these patients.	Exploring patient preferences can be valuable, not only for clinicians who prepare the treatment plan, but also for regulators, when preparing the evaluation file, for informing decisions about reimbursement etc.

→ *We will start with the specific questions for this interview. All questions refer to PPS.*

❖ Questions on design and conduct of PPS

4. In **which steps of the DESIGN of a patient preference study** do you think integrating your knowledge as a patient would be of most value?
    - Defining research question
    - Defining sample
    - Defining exploration or elicitation method
    - Defining instruments (questions, attributes, levels)

5. In **which steps of the CONDUCT of a patient preference study** do you think integrating your knowledge as a patient would be of most value?
    - Participant recruitment
    - Piloting and Data collection
    - Analysis and Interpretation

6. How should patients be involved in a patient preference study; playing **which role** in the participation ladder?

    - Consultation
        - Subject (participating in PPS)
        - Informant (giving/stating their preferences)
    - Advise
        - Advisor (giving advice to the researchers on how a PPS should be designed, conducted)
        - Referent (judging protocols of PPS)
    - Collaboration
        - Research partner (full member of project, participating in meetings on design and conduct of PPS)
        - Interviewer (helping to ask questions on preferences to other patients)
    - Control
        - Client (patient organization can assign what needs to be investigated in a PPS and by who)

7. What **kind of patients** do you think could do 'this job' and **why**?

8. Do patients need any **specific knowledge** or **background**, or can we ask **any patients** to participate?

9. **How much time** would patients be willing to **invest** in helping in the design and conduct of a patient preference study (on a weekly, monthly, yearly base)?

10. Would **compensation be necessary** for a patient who is willing to collaborate in the design and conduct of a PPS?
    - If yes, can you give any **ideas**?

11. Would you as a patient consider **participating** in the design of a patient preference study **yourself**?
    - What would be **incentives** to convince you?
    - What could be **barriers**?

❖ Questions on communication regarding study results

Why would you want to receive results of patient preference studies?

12. Would you be more interested in receiving **general** results after participating in a preference study, **individual** results or your individual results **compared to the average** results?

13. In **what format, in which manner** do you as a patient prefer the results to be presented?

    How would you **understand the most** out of these results?
    (e.g. shortly summarized, very detailed, visually, graphically, verbally, via email, video, personal conversation…)

❖ Round-up Questions

- Did we you have any other **questions** for me?
- Is there something you **want to add**?
- Did we **forgot to ask** something?
- Do you have any suggestions for **other possible interviewees** that might be interested to participate and we can contact?
- Would you feel comfortable to be **contacted again** if we have any follow up questions?

Thank you very much for your time and participation. If you have any questions or remarks, please don't hesitate to contact me.

Supplemental material II: Framework Analysis

Stage 1: Transcription

> ***General explanation and important considerations, see: Gale et al.***
> *A good quality audio recording and, ideally, an at verbatim (word for word) transcription of the interview is needed. For Framework Method analysis, it is not necessarily important to include the conventions of dialogue transcriptions which can be difficult to read (e.g. pauses or two people talking simultaneously), because the content is what is of primary interest. Transcripts should have large margins and adequate line spacing for later coding and making notes. The process of transcription is a good opportunity to become immersed in the data.*

- All the audio recordings were converted into transcripts by IV.
- The interviews were transcribed in their native language (English or Dutch) to keep the original wording. Later, if necessary in the results, translated from Dutch to English.

Stage 2: Familiarization with the interview

> ***General explanation and important considerations, see: Gale et al.***
> *Becoming familiar with the whole interview using the audio recording and/or transcript and any contextual or reflective notes that were recorded by the interviewer is a vital stage in interpretation. It can also be helpful to re-listen to all or parts of the audio recording. In multi-disciplinary or large research projects, those involved in analyzing the data may be different from those who conducted or transcribed the interviews, which makes this stage particularly important. One margin can be used to record any analytical notes, thoughts or impressions.*

- In this study, the interviews were conducted by the same person that made the analysis. Conducting the interviews and analyse them was done in a relative short time frame, which makes familiarization with the interviews much easier.
- Primarily the transcripts were used for familiarization. If the analyser had difficulty in understanding the recording, the analyser re-listened the audio file.
- Each transcript was thoroughly read and re-read to become more familiar with the data.

Stage 3: Coding

- A combined approach was used to classify all data by giving codes: both deductive codes (from specific pre-defines research questions and questions from the interview guide) and inductive codes (from the experiences and views of participants) were given.
- The first 4 transcripts were independently coded by IV and EvO.
- The following coding tree was created after sitting together and discussing the independently coded first transcripts.

Coding tree

1. **Role as patient representative**
2. **Understanding of PP**
   - 2.1. Baseline understanding of PP
   - 2.2. Understanding of PP definition
   - 2.3. Understanding of PPS example
     - 2.3.1 Rheumatic diseases
     - 2.3.2 Neuromuscular disorders
     - 2.3.3 Cancer
3. **Value of patient involvement in stages PPS**
   - 3.1. Research question
   - 3.2. Sample
   - 3.3. Method
   - 3.4. Attributes
   - 3.5. Recruitment
   - 3.6. Data collection
   - 3.7. Interpretation
4. **Patient involvement levels in PPS**
   - 4.1. Consultation
   - 4.2. Advice
   - 4.3. Collaboration
   - 4.4. Control
5. **Requirements for patient involvement in PPS**
   - 5.1. Knowledge
   - 5.2. Time investment
   - 5.3. Compensation
6. **Willingness of patient involvement in PPS**
   - 6.1. Incentives
   - 6.2. Barriers
7. **Communication of study results**
   - 7.1. Importance of results
   - 7.2. Type of results
   - 7.3. Communication format
8. **Opinion of the study**
9. **Patient involvement landscape**

- Gale et al. argue that in this stage, it is valuable to involve other stakeholders to give alternative viewpoints. In our study, we found it sufficient that coding was independently done by two researchers, without actively involving the stakeholders themselves. The reason for this is that involving other stakeholders would have slowed down the coding stage considerably in a tight time frame.

General explanation and important considerations, see: Gale et al.
After familiarization, the researcher carefully reads the transcript line by line, applying a paraphrase or label (a 'code') that describes what he has interpreted in the passage as important. In more inductive studies, at this stage 'open coding' takes place, i.e. coding anything that might be relevant from as many different perspectives as possible. Codes could refer to substantive things (e.g. particular behaviours, incidents or structures), values (e.g. those that inform or underpin certain statements, such as a belief in evidence-based medicine or in patient choice), emotions (e.g. sorrow, frustration, love) and more impressionistic/methodological elements (e.g. interviewee found something difficult to explain, interviewee became emotional, interviewer felt uncomfortable). In purely deductive studies, the codes may have been pre-defined (e.g. by an existing theory, or specific areas of interest to the project) so this stage may not be strictly necessary and you could just move straight onto indexing (stage 5: assigning text to codes), although it is generally helpful even if you are taking a broadly deductive approach to do some open coding on at least a few of the transcripts to ensure important aspects of the data are not missed. Coding aims to classify all of the data so that it can be compared systematically with other parts of the data set. At least two researchers (or at least one from each discipline or specialty in a multi-disciplinary research team) should independently code the first few transcripts, if feasible. Patients, public involvement representatives or clinicians can also be productively involved at this stage, because they can offer alternative viewpoints thus ensuring that one particular perspective does not dominate. It is vital in inductive coding to look out for the unexpected and not to just code in a literal, descriptive way so the involvement of people from different perspectives can aid greatly in this. As well as getting a holistic impression of what was said, coding line-by-line can often alert the researcher to consider that which may ordinarily remain invisible because it is not clearly expressed or does not 'fit' with the rest of the account. In this way the developing analysis is challenged; to reconcile and explain anomalies in the data can make the analysis stronger. Coding can also be done digitally using CAQDAS, which is a useful way to keep track automatically of new codes. However, some researchers prefer to do the early stages of coding with a paper and pen, and only start to use CAQDAS once they reach Stage 5 (see below).

Stage 4: Developing a working analytical framework

General explanation and important considerations, see: Gale et al.
After coding the first few transcripts, all researchers involved should meet to compare the labels they have applied and agree on a set of codes to apply to all subsequent transcripts. Codes can be grouped together into categories (using a tree diagram if helpful), which are then clearly defined. This forms a working analytical framework. It is likely that several iterations of the analytical framework will be required before no additional codes emerge. It is always worth having an 'other' code under each category to avoid ignoring data that does not fit; the analytical framework is never 'final' until the last transcript has been coded.

- All transcripts were now coded using the initial analytical framework, checking if new codes had to be added.
- No new codes were formed. To form the secondary framework, the initial framework was revised to find relations between codes to group them together.
- The final framework consists of codes and categories.

Stage 5: Applying the analytical framework

General explanation and important considerations, see: Gale et al.
The working analytical framework is then applied by indexing subsequent transcripts using the existing categories and codes. Each code is usually assigned a number or abbreviation for easy identification (and so the full names of the codes do not have to be written out each time) and written directly onto the transcripts. Computer Assisted Qualitative Data Analysis Software (CAQDAS) is particularly useful at this stage because it can speed up the process and ensures that, at later stages, data is easily retrievable. It is worth noting that unlike software for statistical analyses, which actually carries out the calculations with the correct instruction, putting the data into a qualitative analysis software package does not analyse the data; it is simply an effective way of storing and organizing the data so that they are accessible for the analysis process.

- We used NVivo 12 software to apply the analytical framework. The final analytical framework (coding tree) was uploaded in NVivo. All the transcripts were coded by IV. In each transcript, sentences and passages of text were highlighted, selecting and attaching an appropriate code from the final coding tree.

Stage 6: Charting the data into the framework matrix

General explanation and important considerations, see: Gale et al.
Qualitative data are voluminous (an hour of interview can generate 15–30 pages of text) and being able to manage and summarize (reduce) data is a vital aspect of the analysis process. A spreadsheet is used to generate a matrix and the data are 'charted' into the matrix. Charting involves summarizing the data by category from each transcript. Good charting requires an ability to strike a balance between reducing the data on the one hand and retaining the original meanings and 'feel' of the interviewees' words on the other. The chart should include references to interesting or illustrative quotations. These can be tagged automatically if you are using CAQDAS to manage your data (N-Vivo version 9 onwards has the capability to generate framework matrices), or otherwise a capital 'Q', an (anonymized) transcript number, page and line reference will suffice. It is helpful in multi-disciplinary teams to compare and contrast styles of summarizing in the early stages of the analysis process to ensure consistency within the team. Any abbreviations used should be agreed by the team.

- NVivo 12 (for Mac) was used to make the framework matrix. The matrix is comprised of one row per case (= participant) and one column per node (= code). The framework matrix was exported to an Excel file.

Stage 7: Interpreting the data

- The data were reviewed by looking at the framework matrix and making connections within and between participants and categories. During the stage of interpretation, the goal is to go beyond the descriptions of individual participants.

Reference: *Gale NK, Heath G, Cameron E, Rashid S, Redwood S. Using the framework method for the analysis of qualitative data in multi-disciplinary health research. BMC Med Res Methodol. 2013 Sep;13:117.*
